# Postgraduate training for trauma prevention, injury surveillance and research, Uganda

**DOI:** 10.2471/BLT.17.200949

**Published:** 2018-04-12

**Authors:** Abdulgafoor M Bachani, Nino Paichadze, Jacob A Bentley, Nazarius Mbona Tumwesigye, David Bishai, Lynn Atuyambe, Stephen Wegener, David Guwatudde, Olive C Kobusingye, Adnan A Hyder

**Affiliations:** a Johns Hopkins International Injury Research Unit, Health Systems Program, Department of International Health, Johns Hopkins Bloomberg School of Public Health, 615 North Wolfe Street, Suite E-8132, Baltimore, Maryland, 21205, United States of America (USA).; b Seattle Pacific University, Seattle, USA.; c Department of Epidemiology and Biostatistics, Makerere University School of Public Health, Kampala, Uganda.; d Department of Population, Family and Reproductive Health, Johns Hopkins Bloomberg School of Public Health, Baltimore, USA.; e Department of Community Health and Behavioral Sciences, Makerere University School of Public Health, Kampala, Uganda.; f Department of Physical Medicine and Rehabilitation, Division of Rehabilitation Psychology and Neuropsychology, Johns Hopkins School of Medicine, Baltimore, USA.; g Makerere University School of Public Health, Kampala, Uganda.

## Abstract

**Problem:**

The burden of trauma and injuries in Uganda is substantial and growing. Two important gaps that need addressing are the shortage of trained people and a lack of national data on noncommunicable diseases and their risk factors in Uganda.

**Approach:**

We developed and implemented a new track within an existing master of public health programme, aimed at developing graduate-level capacity and promoting research on key national priorities for trauma and injuries. We also offered training opportunities to a wider audience and set up a high-level national injury forum to foster national dialogue on addressing the burden of trauma, injuries and disability.

**Local setting:**

The Chronic Consequences of Trauma, Injuries and Disability in Uganda programme was implemented in 2012 at Makerere University School of Public Health in Kampala, Uganda, in conjunction with Johns Hopkins Bloomberg School of Public Health in Baltimore, United States of America.

**Relevant changes:**

Over the years 2012 to 2017 we supported four cohorts of master’s students, with a total of 14 students (9 females and 5 males; mean age 30 years). Over 1300 individuals participated in workshops and seminars of the short-term training component of the programme. The forum hosted three research symposia and two national injury forums.

**Lessons learnt:**

Institutional support and collaborative engagement is important for developing and implementing successful capacity development programmes. Integration of training components within existing academic structures is key to sustainability. Appropriate mentorship for highly motivated and talented students is valuable for guiding students through the programme.

## Résumé

**Problème:**

La charge de morbidité liée aux traumatismes et aux blessures en Ouganda est importante et en augmentation. La pénurie de personnes formées et le manque de données nationales sur les maladies non transmissibles et leurs facteurs de risque en Ouganda constituent deux lacunes importantes qui doivent être comblées.

**Approche:**

Nous avons élaboré et mis en œuvre un nouveau parcours au sein d'un master de santé publique existant, destiné à développer des capacités de niveau universitaire et à promouvoir la recherche sur les principales priorités nationales relatives aux traumatismes et aux blessures. Nous avons également proposé des possibilités de formation à un plus large public et organisé un forum national de haut niveau sur les blessures pour encourager un dialogue national sur la charge induite par les traumatismes, les blessures et le handicap.

**Environnement local:**

Le programme Conséquences chroniques des traumatismes, des blessures et du handicap en Ouganda a été mis en œuvre en 2012 à l'École de santé publique de l'Université Makerere à Kampala, en Ouganda, en collaboration avec l'École de santé publique Bloomberg de l'Université Johns Hopkins à Baltimore, aux États-Unis d'Amérique.

**Changements significatifs:**

Entre 2012 et 2017, nous avons soutenu quatre cohortes d'étudiants en master, pour un total de 14 étudiants (9 femmes et 5 hommes; âge moyen 30 ans). Plus de 1300 personnes ont participé aux ateliers et séminaires du volet formation courte du programme. Le forum a accueilli trois symposiums de recherche et deux forums nationaux sur les blessures.

**Leçons tirées:**

Le soutien institutionnel et l'engagement collaboratif sont importants pour élaborer et mettre en œuvre avec succès des programmes de renforcement des capacités. L'intégration des volets de formation aux structures universitaires existantes est essentielle pour leur viabilité. Un tutorat adapté pour les étudiants motivés et brillants est utile pour les accompagner pendant toute la durée du programme.

## Resumen

**Problema:**

La carga de traumatismos y lesiones en Uganda es notable y sigue creciendo. Dos deficiencias importantes que deben abordarse son la escasez de personal formado y la falta de datos nacionales sobre las enfermedades no contagiosas y sus factores de riesgo en Uganda.

**Enfoque:**

Se ha desarrollado e implementado un nuevo curso dentro de un programa de máster en salud pública existente, destinado a desarrollar la capacidad de nivel de postgrado y promover la investigación sobre prioridades nacionales clave para traumatismos y lesiones. También se ofrecieron oportunidades de formación a un público más amplio y se estableció un foro nacional de alto nivel sobre lesiones para fomentar el diálogo nacional sobre la carga de los traumatismos, las lesiones y la discapacidad.

**Marco regional:**

El programa Consecuencias crónicas de los traumatismos, las lesiones y la discapacidad en Uganda se implementó en 2012 en la Escuela de Salud Pública de la Universidad de Makerere en Kampala, Uganda, junto con la Escuela de Salud Pública Johns Hopkins Bloomberg en Baltimore, EE.UU.

**Cambios importantes:**

Entre los años 2012 y 2017 se respaldaron cuatro cohortes de estudiantes de máster, con un total de 14 estudiantes (9 mujeres y 5 hombres, con una edad media de 30 años). Más de 1300 personas participaron en talleres y seminarios del componente de formación a corto plazo del programa. El foro fue sede de tres simposios de investigación y dos foros nacionales sobre lesiones.

**Lecciones aprendidas:**

El apoyo institucional y el compromiso colaborativo son importantes para desarrollar e implementar programas de desarrollo de capacidades de éxito. La integración de los componentes de formación dentro de las estructuras académicas actuales es clave para la sostenibilidad. Una tutoría apropiada para estudiantes muy motivados y con talento es valiosa para guiar a los estudiantes por el programa.

## ملخص

**المشكلة:**

هناك عبء ضخم ومتزايد ناتج عن الرضوح والإصابات في أوغندا. وهناك فجوتان ينبغي الالتفات لهما، وهما المتمثلتان في غياب الموارد البشرية المدرّبة، ونقص البيانات الوطنية حول الأمراض غير السارية وعوامل الخطورة الناتجة عنها في أوغندا.

**الأسلوب:**

قمنا بتطوير وتنفيذ مسار جديد في إطار برنامج حالي لدراسة الماجستير المعني بالصحة العامة، والذي كان موجهًا لتطوير القدرات على مستوى الخريجين، وتشجيع الأبحاث حول الأولويات الوطنية الرئيسية الخاصة بالرضوح والإصابات. كما قدمنا فرصًا للتدريب إلى جمهور أوسع نطاقًا من المتلقين، وقمنا بإعداد منتدى وطني رفيع المستوى للإصابات بغرض تعزيز الحوار الوطني حول التعامل مع عبء الرضوح والإصابات والإعاقة.

**المواقع المحلية:**

تم تنفيذ برنامج "التبعات المستديمة للرضوح والإصابات والإعاقة في أوغندا" في عام 2012 في كلية الصحة العامة بجامعة ماكيريري في مدينة كامبالا بأوغندا، وذلك بالتعاون مع كلية الصحة العامة بجامعة جون هوبكنز بلومبرغ في بالتيمور بالولايات المتحدة الأمريكية.

**التغيّرات ذات الصلة:**

خلال المدة ما بين عاميّ 2012 وحتى 2017، قمنا بدعم أربع مجموعات من طلاب الماجستير كانت تضم في مجموعها 14 طالبًا (9 طالبات و5 طلاب، بمتوسط أعمار بلغ 30 عامًا). وقد شارك أكثر من 1300 فرد في حلقات العمل والحلقات الدراسية في إطار الشق الخاص بالتدريب قصير المدى في البرنامج. واستضاف المنتدى ثلاث ندوات بحثية ومنتديين وطنيين حول الإصابات.

**الدروس المستفادة:**

هناك أهمية كبيرة للدعم ****من جانب المؤسسات والمشاركة الجماعية لتطوير وتنفيذ البرامج الناجحة لتطوير القدرات. كما أن إدماج عناصر التدريب في الأنظمة الأكاديمية الحالية مهم بدوره لضمان استدامتها. ولذلك فإن التوجيه المناسب للطلاب الموهوبين وذي المستوى المرتفع من الدافعية يمثل عنصرًا قيًما لتوجيه الطلاب في إطار البرنامج.

## 摘要

**问题:**

乌干达的创伤和伤害负担巨大且仍在不断增加。乌干达有两个严峻的问题亟需解决，一是缺乏训练有素的人员，二是缺乏国家层面上关于非传染性疾病及其风险因素的数据资料。

**方法:**

我们在现有的公共健康硕士项目中开发并设置了一个新的分支，旨在培养达到硕士级别能力的人才，并促进对创伤和伤害类国家重点优先医疗项目的研究。我们还向更广泛的受众提供培训机会，并成立了一个高水平的国家级伤害论坛，以促进国家对话，并解决创伤、伤害和残疾的负担。

**当地状况:**

乌干达坎帕拉的马凯雷雷大学公共卫生学院与位于美国巴尔的摩的约翰·霍普金斯大学的布隆博格公共卫生学院合作，于 2012 年实施了一项针对乌干达的创伤、伤害和残疾的慢性后果的项目。

**相关变化:**

2012 至 2017 年间，我们支持了四批学生获得硕士学位，共计 14 名学生（9 名女生和 5 名男生；平均年龄 30 岁）。超过 1300 人参与了该项目短期培训部分的讲习班和研讨会。该论坛举办了三个研究专题讨论会和两个国家级伤害论坛。

**经验教训:**

机构支持和协作参与对于成功开发并实施能力发展课程非常重要。在现有的学术体系中纳入培训内容是可持续发展的关键。为积极主动、有天赋的学生提供适当的指导帮助学生完成课程很有价值。

## Резюме

**Проблема:**

Уганда характеризуется значительным и растущим бременем травматизма. Две важные проблемы, требующие решения, — это нехватка квалифицированных специалистов и отсутствие национальных данных о неинфекционных заболеваниях и их факторах риска в Уганде.

**Подход:**

В рамках существующей программы магистратуры в области общественного здравоохранения авторы разработали и внедрили новое направление, нацеленное на развитие потенциала на уровне выпускников и продвижение исследований по ключевым национальным приоритетам в области травматизма. Авторами были предложены возможности для обучения, ориентированные на более широкую аудиторию, а также был создан национальный форум высокого уровня по вопросам травматизма для содействия национальному диалогу по устранению бремени травматизма и инвалидности.

**Местные условия:**

В 2012 году была создана программа по изучению вопросов хронических последствий травматизма и инвалидности в Уганде на базе Школы общественного здравоохранения Университета Макерере в Кампале, Уганда, совместно с Блумбергской школой общественного здравоохранения Джона Хопкинса в Балтиморе, Соединенные Штаты Америки.

**Осуществленные перемены:**

В течение 2012–2017 годов авторы оказали поддержку четырем когортам студентов магистратуры, в общей сложности 14 студентов (9 женщин и 5 мужчин, средний возраст которых составлял 30 лет). Более 1300 человек приняли участие в тренингах и семинарах по краткосрочной программе обучения. В рамках форума были проведены три исследовательских симпозиума и два национальных форума по травматизму.

**Выводы:**

Институциональная поддержка и совместные усилия имеют важное значение для разработки и реализации успешных программ по развитию потенциала. Интеграция учебных компонентов в существующие академические структуры играет ключевую роль в обеспечении устойчивого развития. Значительную роль в продвижении высокомотивированных и талантливых студентов играет надлежащее наставничество.****

## Introduction

Injuries are a leading cause of death in the African Region, where they claim over 900 000 lives annually,^1^ and within Africa, Uganda has one of the highest burdens.^2^ In 2015, the World Health Organization (WHO) reported over 36 000 deaths and 2460 per 1000 disability adjusted life-years lost from all injuries in Uganda, representing 12% of the total disease burden (20 619 per 1000 disability adjusted life-years) in the country.^1^

As is the case in many low- and middle-income countries, Uganda faces challenges in addressing this burden, due to a shortage of trained people and a lack of national data on noncommunicable diseases and injuries and their risk factors.^3^ Without comprehensive national and disaggregated data on injuries, the magnitude of the burden cannot be appreciated. This poses barriers to defining risk factors and vulnerable groups, as well as assessing the impact of potential interventions. Furthermore, the country lacks a cadre of trauma and injury professionals. Until 2012, there were no graduate-level training programmes focusing on injury prevention, disability assessment and the societal and economic impact of injuries in Uganda; this is an impediment to analytical and operational work in the field. To address these barriers, we developed and implemented a graduate-level training programme aimed at developing such capacity and fostering a national dialogue geared towards concerted actions to reduce the growing burden of injuries in Uganda.

## Local setting

The Chronic Consequences of Trauma, Injuries and Disability Across the Lifespan in Uganda programme was implemented at Makerere University School of Public Health in Kampala, Uganda, in collaboration with Johns Hopkins Bloomberg School of Public Health, Baltimore, United States of America. The programme was funded by the Fogarty International Center of the United States National Institutes of Health in 2012.^4^

## Approach

The programme was designed to integrate within the existing academic structures at Makerere University School of Public Health, so as to enhance its sustainability beyond the funding period. The programme comprised three main domains: long-term training; short-term training and national engagement.

### Long-term training domain

We introduced a new track within the master of public health programme at Makerere University School of Public Health, focused on trauma, injuries and disability. The master programme lasts two years, covering 78 weeks of study, and has both academic and field components. Each credit unit was 15 contact hours, which included lectures, practical work and fieldwork hours. The academic part of the master programme consisted of introductory and specialized courses in public health and included both core courses (total of 30 credit units) and elective courses (3 credit units). In addition, students were required to take track-specific courses (9 credit units) and deliver a seminar series during year 2 (4 credit units). The field component of the programme consisted of two field studies with an output of two field reports (6 credit units), one of which converted into a dissertation research project (9 credit units). Overall, students accumulated a total of 61 credit units before graduating from the programme.

For the track-specific component, three new courses were developed: Chronic consequences of trauma, injuries and disability; Interventions for trauma, injury and disability; and Surveillance and data systems for trauma, injury and disability. Students participated in a two-month advanced training at the Johns Hopkins Bloomberg School of Public Health in Baltimore, United States of America during the recess term of the first year of their master study. This consisted of taught courses in advanced epidemiology and biostatistics and mentoring to help students develop research proposals for their dissertations. Each student was assigned two mentors, one each from Johns Hopkins Bloomberg School of Public Health and Makerere University School of Public Health. The mentors monitored students’ progress in the coursework, identified suitable research projects and gave professional development guidance in the field of injury prevention.

We introduced a two-year postgraduate fellowship, which provided funds for tuition and travel. This kick-started the master programme, generated interest and raised awareness within the student community of the new track and research opportunities in the field of trauma and injuries. Students had to have a degree in health sciences, biological sciences or social sciences or humanities and were recruited from among junior faculty, residents, fellows and staff members in various departments of Makerere University. Applicants were selected based on their academic performance, personal commitment to research on trauma and injury, professional background and previous research experience.

### Short-term training domain

The short-term training aimed to supplement and enhance the long-term training component. This domain was open to a larger audience of postgraduate students and faculty members in Makerere University and externally to staff of other academic institutions, research and private sector organizations working on injuries and disability, and nongovernmental and governmental organizations. Activities included workshops (basic and advanced), symposia and seminars that assisted in training scientists at Makerere University and provided more in-depth, targeted research training to both the master students and other scientists in Uganda. The activities also raised awareness and stimulated dialogue among health and other professionals about the importance of research on trauma and injuries, and fostered collaboration among trauma and injury researchers both within Uganda and internationally.

### National engagement domain

As part of the national engagement domain the programme team established a high-level national injury forum, which led research-to-policy dialogue on trauma and injury in Uganda. The aim was to facilitate the uptake of research into policy and to influence research that would respond to key policy issues. Attendees were university fellows, researchers and faculty, other health and public health professionals, policy-makers and public-sector officials.

## Relevant changes

Over the five years of the programme (2012‒2017), we were able to support four cohorts of students in the master in public health, totalling 14 fellows (9 women and 5 men; mean age 30 years). The programme also supported five independent research projects through a small-grants programme to further stimulate research around key national priorities. The three new master courses in trauma, injuries and disability were co-instructed by faculty from the Makerere University School of Public Health, and Johns Hopkins Bloomberg School of Public Health for three consecutive years, before being taken over by faculty members from the Makerere University School of Public Health. The dissertation research projects of the master students and small-grant recipients covered a variety of injury-related topics and contributed to promotion of research around key national priorities for trauma and injuries in Uganda (Box 1).

Box 1 **Titles of research studies completed by master of public health students and small-grant recipients in the Chronic Consequences of Trauma, Injuries and Disability in the Uganda programme at Makerere University, 2012–2017**

**Completed 2014**

Estimating road traffic injuries in Jinja district using capture–recapture method (Jul–Aug 2014; accepted by *International Journal of Injury Control and Safety Promotion* January 2018)

Incidence and patterns of injuries among children aged 0–5 years living in periurban areas of Wakiso district, Uganda (Aug 2015)

Knowledge, attitudes and practices of commercial motorcyclists on helmet use in Makindye division, Kampala district (Jul–Aug 2014)

Quality of life of persons with physical disabilities receiving community based rehabilitation care in Kayunga district, Uganda (Jul–Aug 2014; published in *Annals of Global Health* in autumn 2017)

**Completed 2015**

Exposure to family domestic violence: an exploration of the experiences and post-traumatic coping mechanisms among medical students at Makerere University (Sep 2015)

Emergency department patient waiting times and its determinants at Mulago national referral hospital (Jul–Aug 2015)

Household coping mechanisms and economic consequences of motorcycle injuries among victims and their households for patients admitted at Mulago hospital (Oct–Nov 2015)

Mobile phone use and the risk of road crashes among commuter taxi drivers in Kampala, Uganda (Sep 2015; submitted to *International Journal of Injury Control and Safety Promotion*; submitted to *BMC Public Health* July 2017)

Outcomes and cost estimates of unintentional injuries among children in a slum community in Kampala (Sep 2015; submitted to *International Journal of Injury Control and Safety Promotion* October 2016)

Pre-hospital care time intervals and associated factors among victims of road traffic injuries in Kampala, Uganda (Jul–Aug 2015; submitted to *International Journal of Injury Control and Safety Promotion* December 2017)

School attendance of 6–18-year-old children with physical disabilities in the Iganga Mayuge health and demographic surveillance site (Jul–Aug 2015)

**Completed 2016**

Alcohol intoxication among *boda boda* drivers, related injuries and health costs at Mulago national referral hospital (Jul–Aug 2016)

Assessment of pre-hospital care provided to road traffic injury patients reporting at Mulago national referral hospital (Jul–Aug 2016)

Determinants of occupational injuries: a case–control study among building construction workers in Kampala city, Uganda (Jul–Aug 2016)

Percutaneous injuries and splash blood exposures among health-care workers in Kampala: knowledge and utilization of HIV and hepatitis B prophylaxis (Jul–Aug 2016)

Utilization of rehabilitation services by human immunodeficiency virus (HIV) patients attending Mulago HIV clinic (Jul‒Aug 2016)

**Completed 2017**

Factors influencing utilization of physical rehabilitation services among injured patients in rural Uganda (Jul–Aug 2017)

Geospacial distribution of pedestrian injuries and associated factors in urban Uganda: a case of Kampala metropolitan (Jul–Aug 2017; submitted to *Accident Analysis & Prevention* February 2018)

The burden of injuries and health seeking behaviours in eastern Uganda – a case study of Iganga-Mayuge health and demographic surveillance site (Jul–Aug 2017)

Notes: Text in brackets shows the date of completion of the dissertation and any scientific publications based on the research. There were 14 students in the trauma, injuries and disability track of the master of public health programme at Makerere University School of Public Health. The small-grants programme supported five independent research projects to further stimulate research around key national priorities in trauma, injury and disability.

During the five years, over 1300 individuals participated in the symposia and seminars in the short-term training component of the main programme. The programme hosted three research symposia (on emergency trauma care; drowning prevention; and emergency medical services) and two Uganda national injury forums (one on the status of injury research in Uganda; the other on practical approaches to injury prevention across various sectors).

## Lessons learnt

The Chronic Consequences of Trauma, Injuries and Disability Across the Lifespan in Uganda programme was developed to respond to needs and priorities for research and training to reduce the burden of trauma and injuries in Uganda. The programme was successful in establishing and running a two-year postgraduate fellowship programme. The main strengths of this component were the highly motivated and talented students chosen through a rigorous selection process; the excellence in teaching and research at both institutions; and the individual mentoring by mentors with a diverse range of interests and experience (Box 2). Through independent research projects, the students were able to apply their knowledge in practice and contribute to the promotion of research around key national priorities for injuries in Uganda.

Box 2 **Summary of key lessons learnt**

- Institutional support and collaborative engagement was important for developing and implementing a successful capacity development programme in trauma, injuries and disability.
- Integration of training components within existing academic structures was key to sustainability of the new master of public health programme.
- Appropriate mentorship for highly motivated and talented students was valuable for guiding students through the master programme.

The founding and incorporation of the trauma, injuries and disability track in the master curriculum was designed to enhance the sustainability of the main programme. Such a model for capacity development had the advantage of making the training programme more accessible and tailored to meet local needs. Furthermore, it enhanced institutional capacity to create a locus for focused research and practice in the field, supporting the interests of both students and faculty members.

The development and successful delivery of three new courses was essential in providing students with a foundation of knowledge in injury research and served as a strong base for the speciality in trauma, injuries and disability. After the initial collaboration with Johns Hopkins Bloomberg School of Public Health faculty members, Makerere University School of Public Health faculty now deliver the courses independently. This demonstrates strengthened capacity for injury research and training at Makerere University School of Public Health and further enhances the programme’s sustainability and impact.

To respond to the need for enhancing writing skills, the programme team implemented a seminar series on scientific writing. We assisted both current students and alumni of the master programme in converting their research projects in scientific publications (Box 1). Through an alumni survey we hope to gather information about our students’ career achievements and other accomplishments of the programme in the future.

Building the skills for conducting, managing and delivering injury and trauma research is important for tackling the growing burden of injuries; yet it is not enough.^5^ We recognize that preventing injuries requires multisectoral collaboration of health and non-health professionals; political, financial and infrastructural investments in data collection, information sharing and viable career paths; strategic use of partnerships and networks; and inclusion of injury in broader public health, health promotion and development plans.^6^
